# Survival of an Antarctic cyanobacterial mat under Martian conditions

**DOI:** 10.3389/fmicb.2024.1350457

**Published:** 2024-04-05

**Authors:** Irene Martin-Andres, Jesús Sobrado, Erika Cavalcante, Antonio Quesada

**Affiliations:** ^1^Departamento de Biología Universidad Autónoma de Madrid, Madrid, Spain; ^2^Lehrstuhl für Biotechnologie, RWTH Aachen University, Aachen, Germany; ^3^Centro de Astrobiología CAB (INTA-CSIC), Madrid, Spain

**Keywords:** Antarctica, cyanobacterial mat, Mars, MARTE, RNAr expression, bacterial community

## Abstract

Antarctica is one of the most outstanding analogs of Mars, and cyanobacterial mats are considered one of the most resilient biological consortia. The purpose of this study is to find out the effect of the Martian conditions on an Antarctic cyanobacterial mat. We exposed an Antarctic microbial mat to Martian conditions in a simulating chamber (MARTE) for 15 d and investigated the variations in the consortium by the use of 16S rRNA gene expression as an indicator of the biological activity. Metabarcoding using the V3-V4 regions of the 16S rRNA gene was used to determine the succession of the active members of the microbial consortium during the experiment. The results showed that the microbial mat, far from collapsing, can survive the stringent conditions in the simulating chamber. Different behaviors were displayed depending on the metabolic capabilities and physiological characteristics of every taxon. The main conclusion is that the Martian conditions did not impair growth in some of the groups, and thus, the investigated Antarctic community would be able to survive in a Martian environment at least during the short experimental period, although elements of the community were affected in different ways.

## 1 Introduction

Mars is considered to be the most similar planet in the solar system to Earth because it has features, such as ice layers and volcanoes, that can be found on our planet (Preston and Dartnell, 2014), and thus, Mars is among the first-order analogs of Earth for astrobiology, with past limnetic systems in the former that could be somehow similar to some aquatic ecosystems in Antarctica (Mikucki et al., 2010). In fact, Earth and Mars shared some similarities in their past, according to Stephen et al. (1999) and data acquired by the *rover Curiosity* in 2013 (Preston and Dartnell, 2014; McKay et al., 2017). Nevertheless, some characteristics of extraterrestrial bodies are not found on Earth nowadays, such as the high levels of UV radiation and extreme temperatures. There are also notorious differences in atmospheric composition and pressure (Preston and Dartnell, 2014). However, on Earth, there are locations that can be considered Mars analogs, such as Arctic and Antarctic bare soils (Table 1), due to the similarity between their temperature ranges (McKay et al., 2017). In general, some microorganisms from the polar regions have been found to persist against simulated Martian conditions (Billi, 2018). Hence, apart from coming from Mars analog environments, microorganisms are used in this discipline, as their adaptations or life strategies could make them capable of surviving in extraterrestrial environments.

**Table 1:** Environmental conditions of Mars, Earth, and Byers Peninsula (Antarctica), according to Bañón et al. (2006) ^(1)^, Britannica (2023) ^(2)^, Schwendner and Schuerger (2020) ^(3)^, Stephen et al. (1999) ^(4)^, and Space (2022) ^(5)^.

	Mars/gas	Earth	Byers Peninsula (Antarctica)
Gas composition	Ar 1.6%, CO 0.08%, CO_2_ 95%, H_2_O 0.13%, N_2_ 2.7% ^(4)^	Ar 1%, CO_2_ traces, 78% N_2_, 21% O_2_, and other gases ^(3)^	Ar 1%, CO_2_ traces, 78% N, 21% O_2_, and other gases ^(3)^
Pressure (kPa)	1.2 Hellas Basin _(impact structure of the planet)_^(3)^	101.3 sea level ^(3)^	95–103.2 ^(1)^
	0.1 Olympus Mons (21.9 Km) ^(3)^	33 Everest (8.48 Km) ^(3)^	
	0.1–1.2 (average 0.7) floor level ^(3)^	120 MPa −11 Km (Marianas Trench) ^(5)^	
Temperature	170–283 K (summer) ^(4)^	288 K (mean) ^(5)^	251–278 K ^(1)^
	226.5 K mean (summer)		270 K (mean) ^(1)^
Humidity	< 35% maximum (summer) ^(4)^	~30% ^(2)^	90% (near lakes) ^(1)^
Radiation	Sun, 550 W/m^2 (4)^	Sun, 1,361 W/m^2^	Sun, 1,361 W/m^2^

Among the most appropriate living forms to perform this kind of study are the microbial mats. As a matter of fact, association among microorganisms in mats has turned out to be a successful strategy in polar environments (Vincent and Quesada, 2012). Microorganisms aggregate by extracellular polymeric substances (EPS), together with proteins, extracellular DNA, lipids, surfactant substances, or pigments. These structures are usually pigmented and are up to several millimeters thick. Microbial mats are typically dominated by cyanobacteria, which are the main primary producers in non-marine polar ecosystems. These are porous structures with channels on which water transports nutrients (de los Ríos et al., 2004). They are even used as trails whereby microorganisms, such as some filamentous cyanobacteria, can move by gliding. Thus, the microbial mat’s physical structure acts as a shelter for the organisms inhabiting it, filtering out a relevant fraction of UVR, keeping more water than the surroundings, and keeping that water in liquid form for longer periods (Vincent and Quesada, 2012). Due to their physical and biological organization, mats serve as a barrier to some harmful characteristics of the external environment (Vincent and Quesada, 2012).

Cyanobacteria are adapted to this environment due to the strategy known as the “lichen strategy,” which consists of maintaining tolerance to several stringent variables (radiation, UV light, desiccation, etc.; Billi, 2017, 2018) instead of developing a large adaptation to only one factor. It is based on applying the dormant state to survive until environmental conditions are appropriate to maintain an active metabolism again (Quesada and Vincent, 1997). Organisms with filamentous morphologies are especially relevant because they not only provide physical structure to the mat but also facilitate providing space to the other organisms and help in attachment to the substrate (de los Ríos et al., 2004). In this manner, while developing the mat, they can function as a refuge and sustainment for heterotrophic microorganisms (such as those belonging to *Actinobacteria*, *Bacteroidetes*, *Firmicutes,* and *Proteobacteria* Phyla). In addition, the mat structure is beneficial to organisms from higher trophic levels as some metazoans (Makhalanyane et al., 2015). Thus, cyanobacterial mats have a key role in biodiversity maintenance (Vincent and Quesada, 2012). In fact, it is widely recognized that cyanobacteria are ecologically crucial in supporting polar environments as the most abundant primary producers in non-marine ecosystems in Antarctica (Makhalanyane et al., 2015).

Dryness is one of the main threats these communities must face because accessibility to liquid water is among the main requisites to conduct microbial activity. Some cyanobacteria, like *Nostoc*, display tolerance against desiccation (Billi et al., 2019), quickly restarting their activities when it is hydrated after long periods of dehydration. However, other cyanobacteria, such as *Phormidium,* require longer periods of rehydration (Vincent and Quesada, 2012). Other organisms can generate specialized structures (such as spores, seeds, or akinetes) to maintain anhydrobiotic metabolisms (Billi, 2017). Among cyanobacteria, *Chroococcidiopsis* can resist both desiccation and radiation (Billi, 2017, 2018). Moreover, to survive, life on Mars should face more threats in addition to the lack of liquid water, or high radiation, such as the low pressure, low temperature, and content in atmospheric gases different from those found on Earth (i.e., extremely low oxygen pressure; Table 1). This is the purpose of MARTE and other extraterrestrial simulators in which environmental conditions can be controlled. In our study, we used MARTE, one of the 14 simulators in the world, which allows programming a series of environmental conditions: gas mixture, Martian dust, windstorm, pressure, temperature, humidity and hydration, PAR, and UV radiation (Sobrado et al., 2014, 2015).

According to the information gathered by Schwendner and Schuerger (2020), there exist living forms capable of not only surviving but also growing while being exposed to Martian conditions. These are the cases of *Anabaena* sp., *Clostridium* sp., or *Synechococcus* sp.

Considering that cyanobacterial mats represent a successful strategy in Mars analog environments, we hypothesized that Martian conditions should not have an effect on the structure of the community. The mat should be capable of surviving in this situation. In order to solve this inquiry, we configured environmental factors (Table 2) in the simulation chamber MARTE (Sobrado, 2020), so that they were as close as possible to real conditions on Mars (Table 1). In addition, instead of supporting either survival or growth based on counting cells/spores or methane production in the case of archaea (Schwendner and Schuerger, 2020), in this study, we have used the production of mRNA for the 16S rRNA gene as an indicator of biological/metabolic activity and have sequenced the mRNA to reveal their identity.

**Table 2:** MARTE’s range of environmental variables during hydrological cycle experiments.

MARTE	Daytime	Interphase	Nighttime
Pressure (kPa)	2	0.1	2
Temperature (K)	283	253	258
Relativity Humidity (%)	35	-----	10
Hydration (eq. resistance, Ohm)	250^*^(<733)	993	993

Every sample received 6 h of light (UV and VIS radiation) and 18 h of darkness for periods of 24 h, except for the very first one sample (8 h), which received 6 h of light and 2 h of darkness. ^*^All samples at first day, completely hydrated (Hydration < 250 Ω). Throughout each day, hydration decreases, and therefore, the equivalent resistance increases as the sample dries to its maximum at nighttime. During the daytime, the equivalent resistance fluctuates except for the first one with values less than 733 Ohm.

Thus, this study aims to find out the effect of the Martian conditions on an Antarctic microbial mat from Byers Peninsula (Livingston Island, South Shetland Islands, Antarctica), investigating what organisms, from a complex consortium, can proceed with their metabolic activity along an exposure of 15 d to the assayed conditions, using the MARTE simulation chamber.

## 2 Materials and methods

### 2.1 Field sampling

The microbial mat submitted to Martian conditions was obtained in 2013 from a swamped area in Byers Peninsula, one of the protected areas of Antarctica, with the official permit of the Spanish Polar Committee. This region is the *Antarctic Specially Protected Area* (ASPA) #126. It is located in the western part of Livingston Island, near the International Byers Camp (South Shetland Islands, Antarctica, 62° 34′ 35″–62° 40′ 35″S, 60° 54′ 14″–61° 13′ 07″W; Bañón et al., 2006; Quesada et al., 2009). The area had a permanent layer of water of 5 cm deep during the sampling period. The sample, which had a surface area of 10 × 10 cm and was visually homogeneous, was collected with a sterile stainless steel spatula. Immediately, the sample was introduced into a sterile Whirl-Pak plastic bag and frozen at 253 K in the field. Afterward, it was transferred to 193 K until the moment when the experiment would take place.

### 2.2 Revival and adaptation of microbial mats before the Mars simulation

Four weeks before the exposure of the biological material to MARTE, the sample was gently thawed at room temperature in a 9-cm-diameter sterile Petri dish, sealed with parafilm, and then incubated at 277 K in a climatic chamber (Climacell 2.2, Fisher) with a photoperiod of 6:18 h (light: dark) at a maximum photon flux fluency of approximately 50 μmol photon m^−2^ s^−1^ from which 20.29 μmol m^−2^ s^−1^ corresponded to photosynthetic photon flux density (PPFD) provided by fluorescent light (DPRO MITW 12 W/840 E27, by OSRAM). The mat was maintained in water saturated with sterilized MilliQ water for 4 weeks. No culture medium was added to the microbial mat. These conditions were maintained to reactivate the metabolism of the organisms from the mats before being introduced into the MARTE chamber.

### 2.3 Simulation chamber and performance

The MARTE chamber simulated conditions like those registered on Mars. MARTE had a gas composition of 100% CO_2_ (95% in Mars), its pressure (0.1–2 kPa) was slightly higher than in Mars (0.1–1.2 kPa), and both its temperature range (253–283 K) and humidity (10%–35%) were inside the range in Mars (170–283 K, <35%). Radiation in MARTE was slightly higher (666 W/m^2^; equivalent to PPFD; photosynthetic photon flow density; of 290.41 μmol m^−2^ s^−1^) than in Mars (550 W/m^2^; Table 1; Sobrado, 2020).

The MARTE chamber, the sensor devices, and the experimental procedure are described in detail in Sobrado (2020). It delivers water to the biological material automatically to keep constant humidity and hydration in the material in three different periods (daytime, interphase between day and night, and nighttime). We have recreated the environmental daytime/nighttime variations that correspond to the SOL on Mars (a Martian day, similar to the Earth with 24 h and 37 min). The daytime period is a quarter of the nighttime period, mimicking some latitudes and seasons on Mars (Table 2).

Regarding the water cycle in MARTE, in the daytime, there is mainly condensation and evaporation, and in the nighttime, there is freezing and sublimation. In the special case of the interphase between daytime and nighttime, there is evaporation and freezing, and in the interphase between nighttime and daytime, there is thawing and evaporation.

The experiment in MARTE started with the pressure at 2 kPa of CO_2_, 283 K, and radiation source in the sample holder. The maximum radiation was reached 15 min after the source was turned on, simulating a sunrise. After 6 h, we have the interphase period. In the interphase between daytime and nighttime, we quickly decreased the pressure down to 0.1 kPa producing rapid freezing of the sample from top to bottom at 253 K, at a rate of 35 s per Kelvin degree or 0.03 K/s, which is maintained with the cryostat at a temperature of 258 K that simulates nighttime on the red planet.

In MARTE, we used two methods to cool the sample. We cooled the sample by conduction from below by extracting heat with the sample holder with a glycol circuit and from above by convection or absorption when pumping the vacuum chamber at a maximum speed. Thus, we created a layer of superficial ice that protects the sample against external radiation and minimizes the loss of humidity due to evaporation, mimicking the natural conditions on Mars.

Radiation control is carried out by turning the Xenon source on and off. Relative humidity changes rapidly with the temperature decrease in the nighttime cycle. The appearance of ice by absorption and the constant temperature of 258 K decreases the relative humidity up to the threshold value (<10%) at 2 kPa pressure on MARTE. In the 6 h daytime cycle, at dawn, the sample holder temperature was increased up to 283 K with a ratio of 1 K/min, and the Xenon source was turned on. As the temperature rose, the ice melted, and the water started evaporating. This is the point where we began to recreate the condensation through water injections, mimicking the minimal hydration and relative humidity conditions that favor the Antarctic microorganisms.

MilliQ water is provided by an inner pulse valve in the vacuum chamber. An external 15-liter-capacity reservoir ensures both the availability and maintenance of differential pressure toward the vacuum chamber. Relative humidity is quantified by a Honeywell HIH-400 series sensor and hydration by a homemade sensor. To monitor the gases in the chamber, including water, an RGA (quadrupole mass spectrometer) is used. Solar radiation is simulated in MARTE using a Xenon source (Hamamatsu 150 W L11033; Table 3). This lamp produces a spectrum in the range between 185 and 2,000 nm. Pressure is controlled by a Pfeiffer DUO 20 rotative vacuum pump with a gas dose valve (RME 005 A by Pfeiffer) with variable conductance, which also regulates the inlet CO_2_ flux. MARTE works or pumps dynamically. Temperature is measured in the sample holder and in the bottom of the Petri dish, where the sample is placed, by a K-type thermocouple. Atmospheric temperature at a few cm above the sample is quantified by an RTD Pt100 class A temperature sensor. Glycol cryostat provides cold and heat, if necessary, to maintain the stability of the sample’s support during the day and night. This is possible due to a close glycol circuit and the control of a 280 W resistance.

**Table 3:** Radiation conditions configured in MARTE.

*Light (6 h of light, 18 h of darkness)*	UVA	320–395 nm	1.46 W/m^2^	*Radiation (source: Xenon; 185 nm-2,000 nm)*	666 W/m^2^ (PPFD: 290.41 μmol m^−2^ s^−1^)
	UVB	265–322 nm	0.33 W/m^2^		
	UVC	225–280 nm	0.25 W/m^2^		

PPFD, Photosynthetic Photon Flux Density (400–700 nm).

### 2.4 Exposure procedure

The experiment took place in the years 2019–2020. The mat fragment (3 cm by 3 cm) was exposed to the conditions for a given period in MARTE and then was removed from the chamber, and two cores of 8 mm diameter were taken at random with metal sterile corers, avoiding the edges of the 3 × 3 cm sample, which are considered replicates. Then, another ‘revived’ fresh fragment of the mat was inserted into MARTE for the next period. This procedure was repeated for nine different exposure times: 0 h (T0) (acting as a control), 8 h (8 h), 16 h (16 h), 24 h (24 h), 3 days (3 d), 5 days (5 d), 10 days (10 d), and 15 days (15 d).

### 2.5 mRNA extraction, amplification, and sequencing

Once each mat fragment had been submitted to Martian conditions for the time required, MARTE was stopped, and pressure equilibrated with the external atmosphere. It takes 6 min from opening MARTE until the sample can be stored in the freezer at −80°C. Samples were then kept at −80°C without any extraction or modification to preserve the cellular components. For RNA extractions, the 8 mm cores from each exposure time were thawed at room temperature, and three small cores of 1 mm diameter were obtained at random with the mouth of a sterile syringe and mixed together in approximately 200 mg of sample. mRNA was extracted with RNA PowerBiofilm® extraction kit, following the instructions of the manufacturer, in an RNAase-free laboratory at *Parque Científico de la Comunidad de Madrid*. DNAase was employed to remove the remaining DNA. Then, reverse transcriptase was used to produce cDNA. The V3-V4 region from the 16S rRNA gene was amplified from the cDNA using universal 16S primers 341 F (5′-CCTAYGGGRBGCASCAG-3′) and 806 R (5′-GGACTACNNGGGTATCTAAT-3′). Then, barcodes and adapters were added to the purified DNA to perform the sequencing process with Illumina MiSeq. This region includes sectors of V4, which is specially preserved in the bacteria domain (and is effective in identifying *Cyanobacteria* Phylum), and the V3 region, which is very changeable, thus it allows to differentiate taxa at different levels (Zhang et al., 2018).

Qiime2-2021.2 version (Qiime2docs, 2021) was the program whereby the reads obtained from the sequencing process were analyzed. *Amplicon sequence* var*iants* (ASVs) are, unlike *operational taxonomic units* (OTUs), not only exact sequences but also reproducible (Maruyama et al., 2020). This was considered crucial in this experiment. The analysis protocol was followed (Maruyama et al., 2020). ASV establishment was performed by the DADA2 *plugin* (Maruyama et al., 2020), which can determine ASVs from the sequence quality data. This plugin covers various processes to determine ASVs. It both filters and cuts the sequences depending on their quality, establishes an error pattern, determines the lectures with distinctive sequences, joins the *paired ends,* and removes chimeras. ASVs obtained were taxonomically assigned using Greengenes. The taxonomical adscription in cyanobacteria taxa was coincident with the microscopical observations of the microbial mat (data not shown).

### 2.6 Statistics and ecological analyses

With the results obtained by bioinformatic approaches, the differences among the communities exposed to different periods in terms of diversity and ecological succession were investigated.

Concerning the statistical analyses, the data provided by Qiime2 did not have a normal distribution, so statistical tests for non-normal samples were the ones used in this study. The similarity between the communities under different exposure periods was evidenced with *non-metric multidimensional scaling* (NMDS; Alan, 2016), using the *vegan* package (Oksanen et al., 2020) in R. NMDS was interpreted in this study as an exploratory analysis aimed at observing the evolution of the Antarctic mats through time.

We investigated the significant differences among samples (*p* < 0.01) using R software (R-project, 2022). Normality was tested by Shapiro tests in R (R-project, 2022). The statistical correlation between each pair of samples was verified with the Wilcoxon test (R-project, 2022). We performed a Heatmap (using the *ampvis2* package (Andersen et al., 2018) in R) with the aim of identifying the most abundant taxa and how they varied through time. The most relevant taxa were identified (in abundance terms), and their ecological role was studied.

## 3 Results

**Figure 1:**
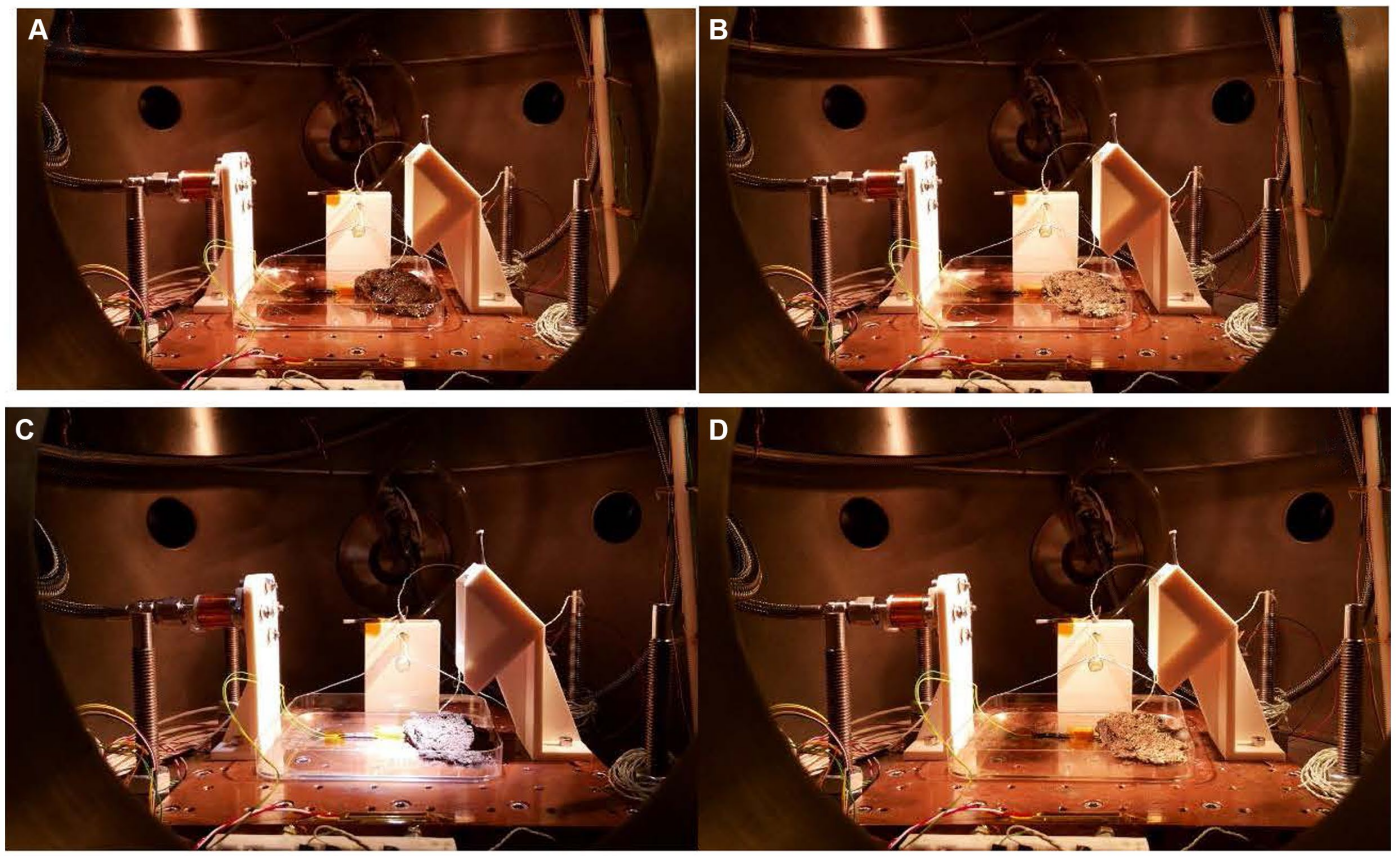
**(A)** First day before starting the experiment. Hydration 250 Ω. **(B)** First day after interphase time. Hydration 993 Ω. **(C)** Tenth day before interphase time. Hydration 733 Ω. **(D)** Fifteenth day after interphase time. Hydration 993 Ω. The distance between the holes in the sample holder is 25 mm.

The macroscopic changes were monitored by photographs taken during the 15d exposure (Figure 1). Figure 1A shows the sample in the Mars daylight conditions. The sample has the maximum possible hydration (250 Ohm) after 4 weeks in the climatic chamber sealed with parafilm. At this point, the vacuum had started, so on the first day, the excess of liquid water was quickly evaporated, and the water microinjection cycle started to simulate the Mars relative humidity and hydration at daylight. The system is pumping continuously to maintain 20 mbar in the chamber. Thus, the atmospheric conditions of Mars are imitated. Figure 1B shows the sample after a 6 h simulation inside the MARTE vacuum chamber. The hydration is minimum, the sample is frozen, and the equivalent resistance is maximum (993 Ohm). The sample freezes from top to bottom by heat absorption due to the vacuum pump in the interphase time (Sobrado, 2020; see Supplementary Video 1). The outside surface is dry, and the surface color is paler due to the ice crystals produced during the interphase. On the tenth day (Figure 1C), the sample continues to lose hydration and volume day by day, and part of it is restored in each hydrological cycle. The sample had the minimum level of hydration (733 Ohm) and humidity in the Martian conditions after 10 d in the Mars environment. The temperature of 283 K helps to maintain a good level of hydration. The picture taken on day 10 indicates that the microbial mat was stable with the hydration cycles allowing conservation of the shape and aspect. On day 15 (Figure 1D), the picture shows the sample after nighttime just before opening MARTE to air and before storing the sample in the freezer at 193 K, until the moment to perform the biological analysis. The color and shape of the microbial mats were slightly affected by the 15-d exposure to MARTE.

With respect to the sequence analyses, 2,301,701 forward and reverse reads were obtained from the bioinformatic approach. After the application of the DADA2 plugin, 1,376,768 reads remained with a standard quality of over 30. From these reads, DADA2 identified 4,478 ASVs with an average length of 414.5 nucleotides. The Greengenes database was used to assign a taxonomical identification (at 99%) to each ASV. The rarefaction curves (Supplementary Figure 1) indicated that diversity was complete with 25,000 reads and that number was used for the analyses.

**Figure 2:**
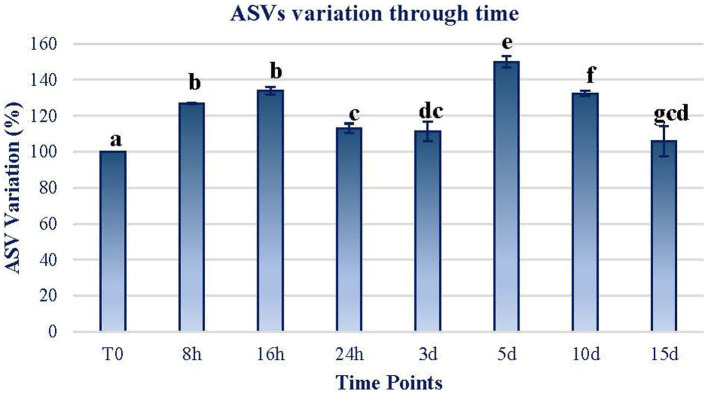
ASV variation through time. The variation in terms of the percentage of the ASVs counted in each sample in reference to the initial sample (T0) is plotted. The data of each sample time are obtained by calculating the mean between the values of the two replicates of each time sample. Error bars represent the range of variation at each time point. The same letters indicate no significant differences at *p* < 0.05 (Wilcoxon test).

The first effect of the exposure to MARTE conditions was related to the variation in the amount of ASVs through time. As can be observed in Figure 2, the amount of ASVs increased significantly (Wilcoxon test; *p* < 0.05) on a short-term basis. In the range between 24 h and 3 d, the number of ASVs declined although they were not significantly different among them (*p* > 0.05). The ASV quantity increased significantly again in the range at day 5. However, after this point, the number of ASVs dropped to almost the initial levels.

**Figure 3:**
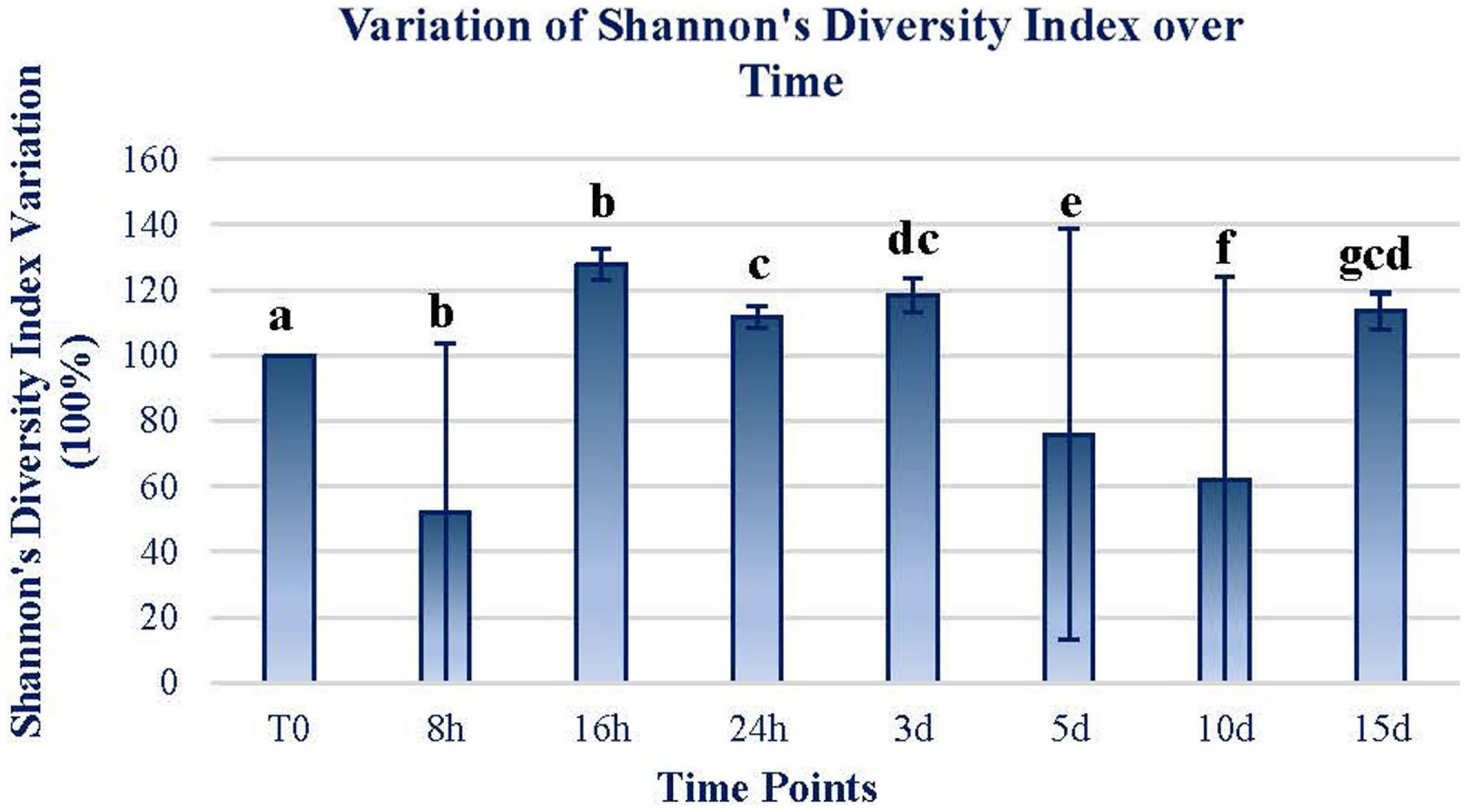
The variation of Shannon’s diversity index through time. The figure expresses the variation in terms of the percentage of Shannon’s diversity index from the original sample. The original value at T0 was 4.35. The data of each sample time are obtained by calculating the mean between the values of the two replicates of each time sample. Error bars represent the range of variation at each time point. The same letters indicate no significant differences at *p* < 0.05 (Wilcoxon test).

Shannon’s diversity index of the mats exposed for the different periods follows a similar trend, with significant increases in the short term and then decreases significantly. At the end of the 15-d exposure period, the diversity index increased by 15% in relation to the initial diversity (Figure 3). There are only two time periods (16 h and 5 d) in which Shannon’s diversity index increases remarkably, by more than 30%. In all the other time periods, the diversity index is slightly higher than before the exposure period.

**Figure 4:**
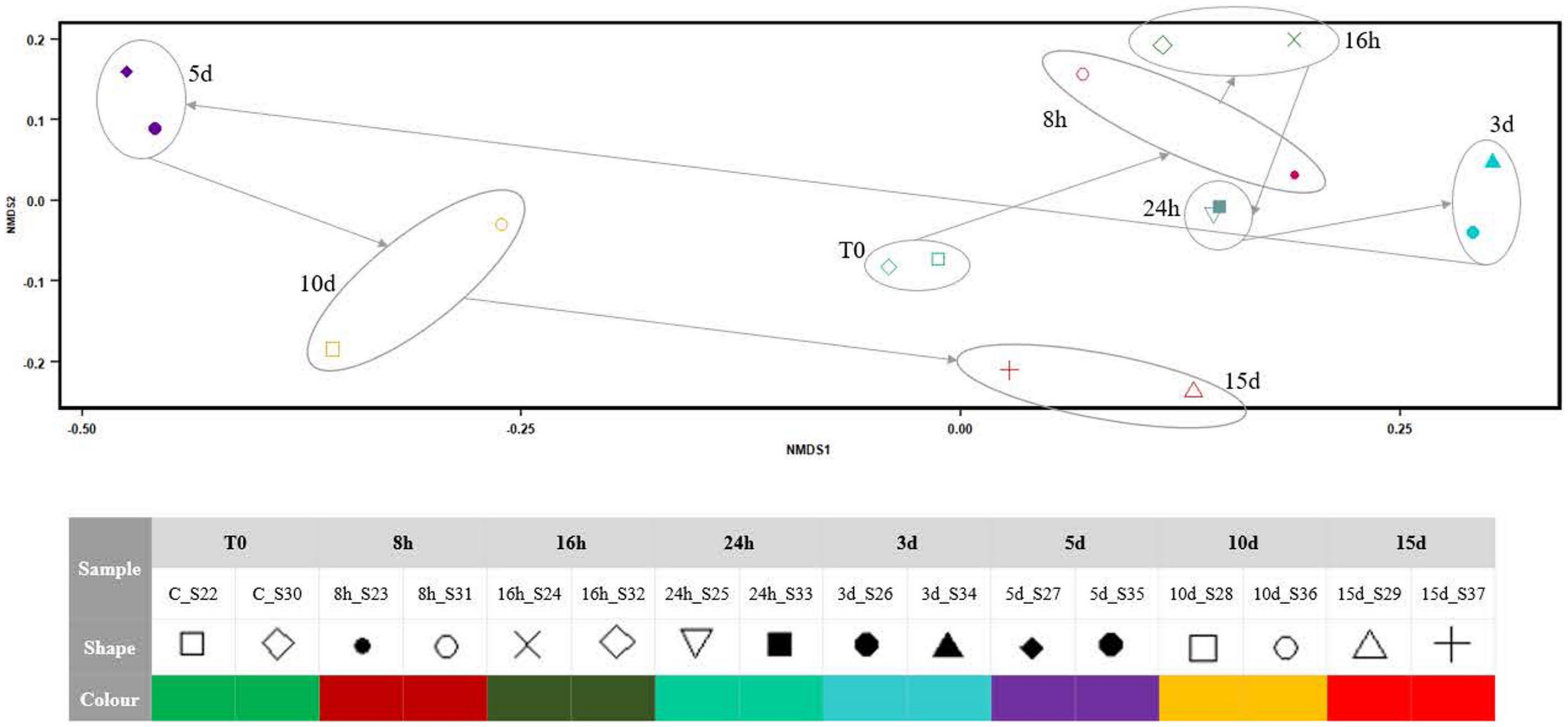
NMDS plot showing the differences in the community structure after exposing the Antarctic microbial mat to different times of Martian condition. The stress of the analysis was 0.08284.

NMDS (Figure 4) indicated that the microbial mat community structure, as 16S RNA gene expression, showed small variations from the onset of the Martian conditions during the first 24 h. From day 3, the 16S RNA gene expression of the community changed markedly in the x-axis, with a relevant breakpoint from day 3 to day 5. During the next 10 d, the microbial mat expression seems to go back to the initial state along the x-axis, although the recovery is not complete as per the y-axis.

**Figure 5:**
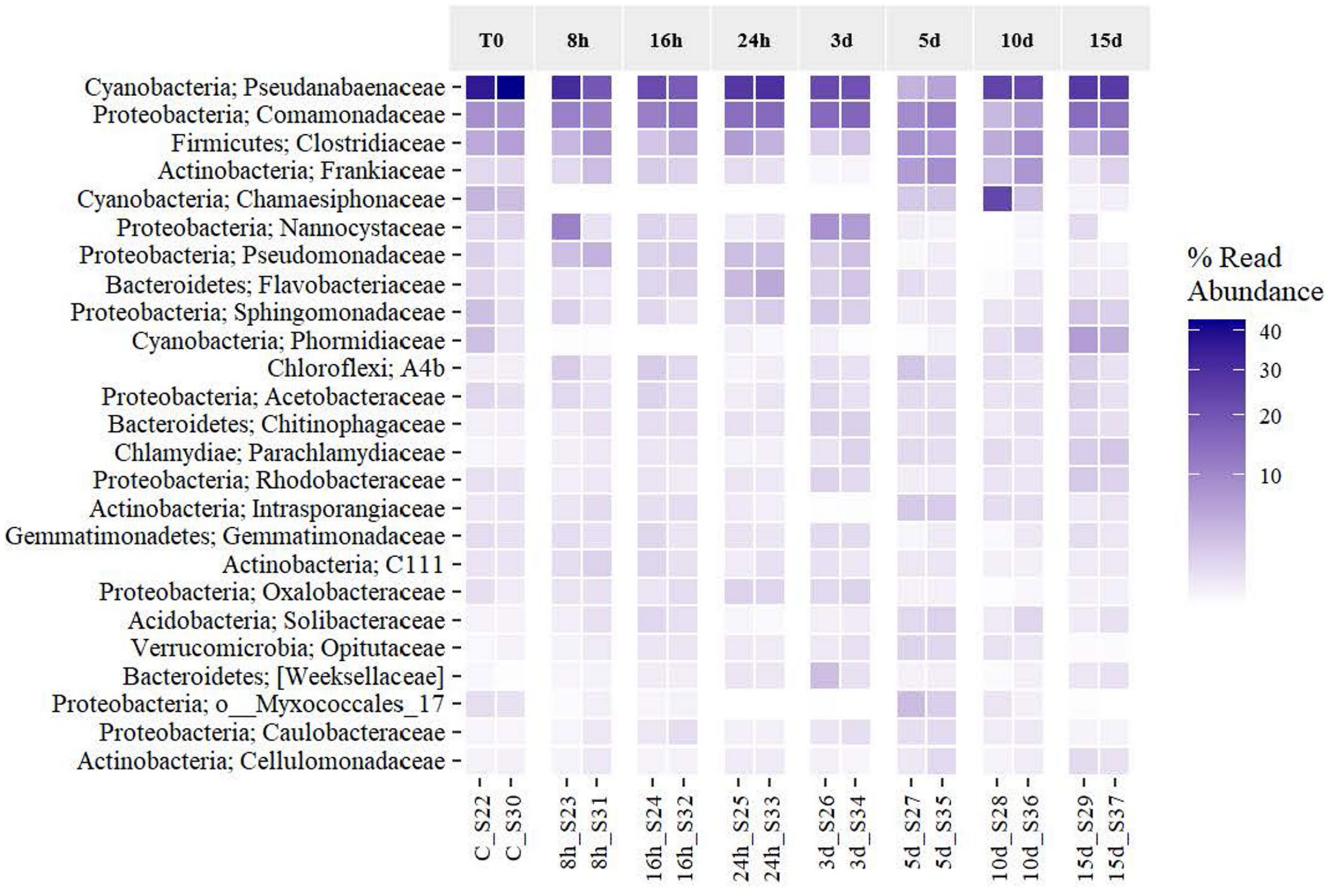
Effects of Martian conditions on the relative abundance of the activity of the most frequent taxa (at the phylum and family level if available) in the Antarctic cyanobacterial mat through time.

An analysis of the effects of the exposure to Martian conditions on the relative abundance of the most frequent taxa (to the highest taxonomic resolution possible) was performed (Figure 5). The six more frequent taxa placed at the heatmap, assigned to the families *Pseudanabaenaceae* (40.05% on T0 and 26.92% on day 15), *Comamonadaceae* (8.58%–14.21%), *Clostridiaceae* (6.14%–6.25%), *Frankiaceae* (1.45%–1.27%), *Nannocystaceae* (1.53%–0.68%), and *Chamaesiphonaceae* (3.83%–0.41%) covered together on average 49.22% of the total abundance of the microbial mat (61.57%–49.75%). Even with the longer exposure to Martian conditions, all taxa were expressing their 16S rRNA gene, although in different relative abundances. Figure 5 reveals that the dominant taxa frequency of expression was stable until day 3. At this point, they started to decrease until day 10, when they seemed to have recovered their original abundance. In the case of the *Comamonadaceae* and *Clostridiaceae* families, they were even more abundant at the end of the experiment. The rest of the taxa, which had a percentage abundance below 20% at T0, increased as time went by. Some taxa, such as *Sphingomonadaceae and Phormidiaceae,* increased at the end of the exposure to MARTE and reached the highest frequency that was found at the beginning of the experiment. Regarding the phylum level, the original mat was dominated by *Cyanobacteria*, *Proteobacteria*, *Firmicutes*, and *Actinobacteria*, but Martian conditions seemed to benefit the growth of other phyla such as *Planctomycetes* during the first 3 d and *Bacteroidetes* at the last day. From Figure 5, Martian conditions led to the reorganization of the mat’s community structure of the active elements. While the original structure was pyramid-shaped in which the system was dominated by the activity of a few types of organisms, the community subjected to the longer exposure presented a more balanced dominance, with more taxa becoming actively dominant.

To shed light on the ecological changes happening in the mat after exposure to Martian conditions, the relative abundance changes occurring with the most abundant taxa were investigated. The sample with a higher number of different ASVs above 1% relative frequency showed 17 ASVs. Thus, to normalize the samples, we took the first 17 ASVs of each sample to carry out the ecological analysis. These 17 ASVs represented, on average, 47.43% of the total reads in the mat, which was considered a representative portion of the total biodiversity. The abundance data were then regrouped according to the taxonomic assignment of each ASV, and those belonging to the same taxon were regrouped. Finally, and considering each set of 17 ASVs belonging to each sample, we summed up 32 different ASVs. The following analyses referred to these 32 taxa, except for six of them, because we were not able to provide an ecological inference for their taxonomic assignment.

**Figure 6:**
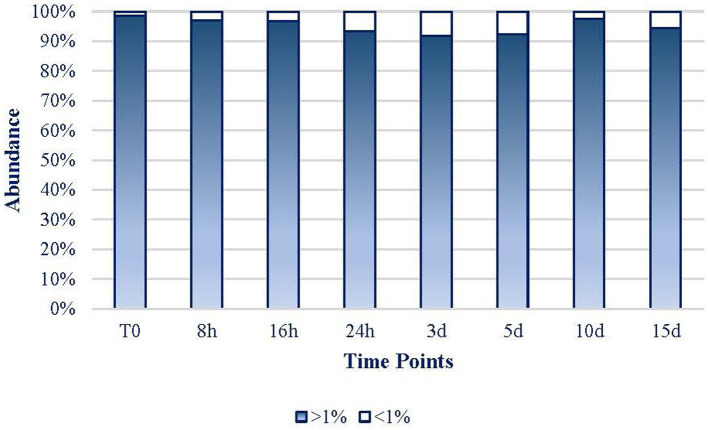
Evolution of the dominant > 1% abundance (17 ASVs per sample; white bars) and the rare (<1% abundance; blue bars) taxa.

As it can be inferred from Figure 6, the expression of the 17 dominant taxa (defined as >1% reads) of each sample maintained their role throughout the experiment representing at every period more than 91.8% of the mat community’s expression. Only on day 3, rare taxa (less than 1% of reads abundance) represented more than 8% of the abundance in the mat. Hence, the 32 more frequent taxa (17 ASVs per sample) in the experiment were considered to be 100% of the mat community to facilitate the rest of the analyses. Hereafter, every percentage data were referred to these 32 taxa that will be considered as the whole mat.

**Figure 7:**
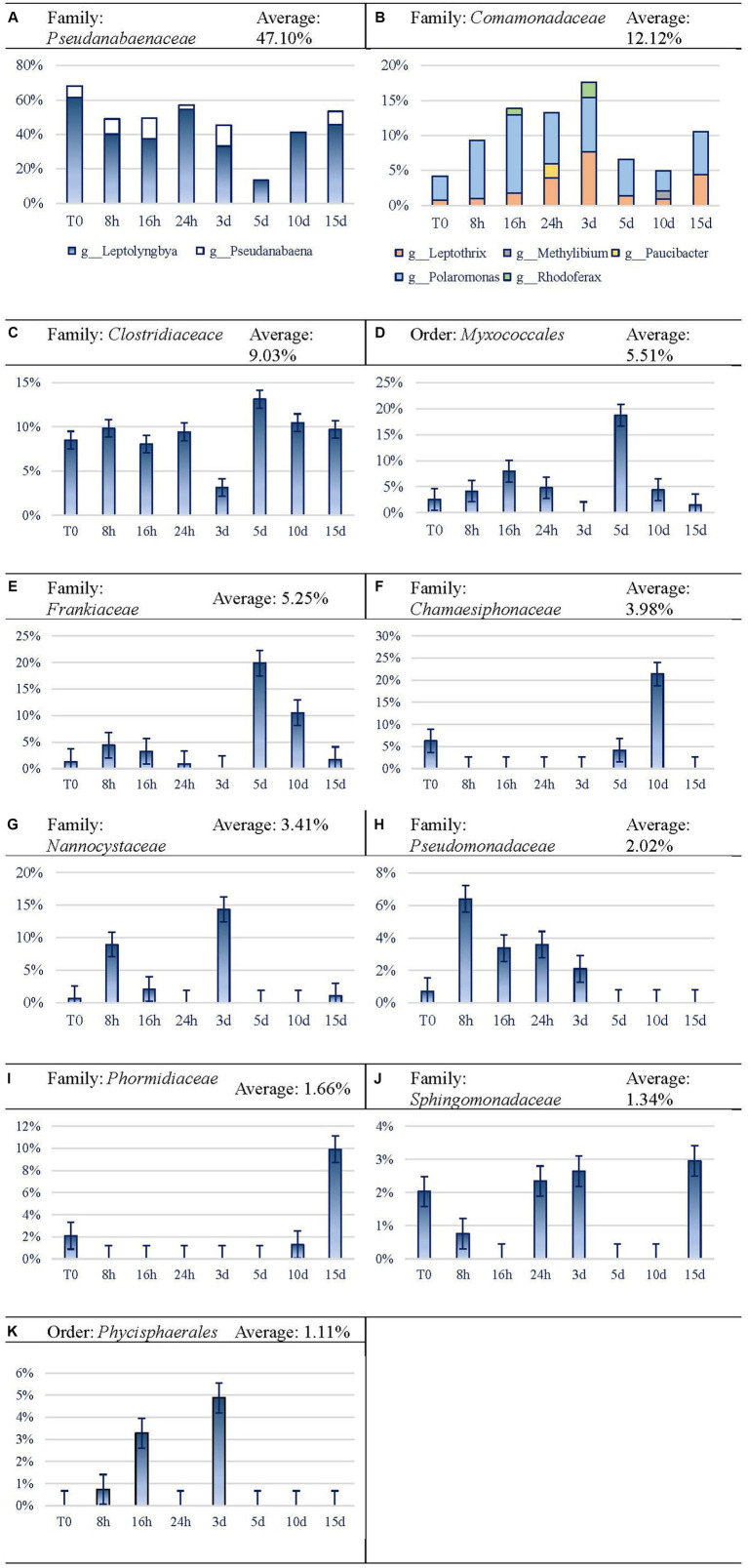
Evolution of the frequent (>1%) taxa activity during the 15-day experiment. For easier readability, the axis legend has been omitted. x-axis is “Time Points,” while y-axis is “Variation.” **(A)** Family: Pseudanabaenaceae, **(B)** Family: Comamonadaceae, **(C)** Family: Clostridiaceace, **(D)** Order: Myxococcales, **(E)** Family: Frankiaceae, **(F)** Family: Chamaesiphonaceae, **(G)** Family: Nannocystaceae, **(H)** Family: Pseudomonadaceae, **(I)** Family: Phormidiaceae, **(J)** Family: Sphingomonadaceae, **(K)** Order: Phycisphaerales.

In this analysis, we used the family taxonomic level as the basis, and when taxonomic resolution was allowed, the genus level was also considered. Dominant taxon variation is shown in Figure 7. *Cyanobacteria* were the most abundant taxa at the phylum level, reaching an average of 52.74% of reads. Among them, *Pseudanabaenaceae* represented 68.18% at the beginning of the experiment, reducing its relative expression until reaching the minimum expression on day 5 by 5-fold. Afterward, the expression of this group increased until the end of the experiment, although it did not reach the initial proportion. Regarding this family, *Leptolyngbya* is the most abundant one and leads the recovery of the family from 5 d on, while *Pseudanabaena* is noticeable throughout the first 3 d and on day 15 (Figure 7A). *Comamonadaceae* (Figure 7B) and *Clostridiaceae* (Figure 7C) seem to be favored by Martian conditions within the microbial mat, as they increased their relative proportion after longer exposure to MARTE entailing a presence of 12.38 and 9.70%, respectively, at the end of the experiment. In the case of *Comamonadaceae*, which follows an inverse trend to *Pseudanabaenaceae*, five genera and a sixth taxon (whose taxonomic resolution did not reach the genus level) have been noticed. *Polaromonas* is the most remarkable among the five genera, because it is the only one noticed every time and it is, by average, the most abundant one (6.52%). *Leptothrix* is the second genus in importance, although it is not as abundant (2.73% average) as *Polaromonas*, but it does appear in every time sample. *Myxococcales* Order (Figure 7D) and *Frankiaceae* family (Figure 7E) shows erratic behavior, as their expression increases for the first 16 h and decreases between 1 and 3 d. On day 5, they both raised their expression levels and again, after that time, they got their abundance reduced to similar values to the original ones in the case of *Myxococcales* or lower than the first ones in the case of *Frankiaceae*. *Chamaesiphonaceae* (*Cyanobacteria*) became very abundant on day 10 (Figure 7F), although its relative abundance became much lower at the end of the experiment. Nevertheless, the high differences in the replicates (37.70 and 5.16% for 10 d replicates) may indicate that a large colony of this organism was incorporated at random in one of the replicates of this analysis. *Nannocystaceae* (Figure 7G) case is similar to the previous ones, as it experiences increases and decreases, although it practically stops its expression at day 3. *Pseudomonadaceae* family (Figure 7H) seemed to benefit from Martian conditions, growing enormously in the first hour and then starting to decline until day 3 when the relative expression was even lower than at the beginning of the experiment. *Phormidiaceae* showed a high increase in activity at the end of the experiment (Figure 7I). The expression of *Sphingomonadaceae* (Figure 7J) did not show a clear relation with time, decreasing for the first 16 h and then regressing their expression to the initial level between 1 and 3 d. *Phycisphaerales* Order (Figure 7K) shows similar behavior to *Chamaesiphonaceae*. The taxon increases activity at concrete time periods between 8 and 16 h, and at 3 d, but after that point, it declines and never recovers.

**Figure 8:**
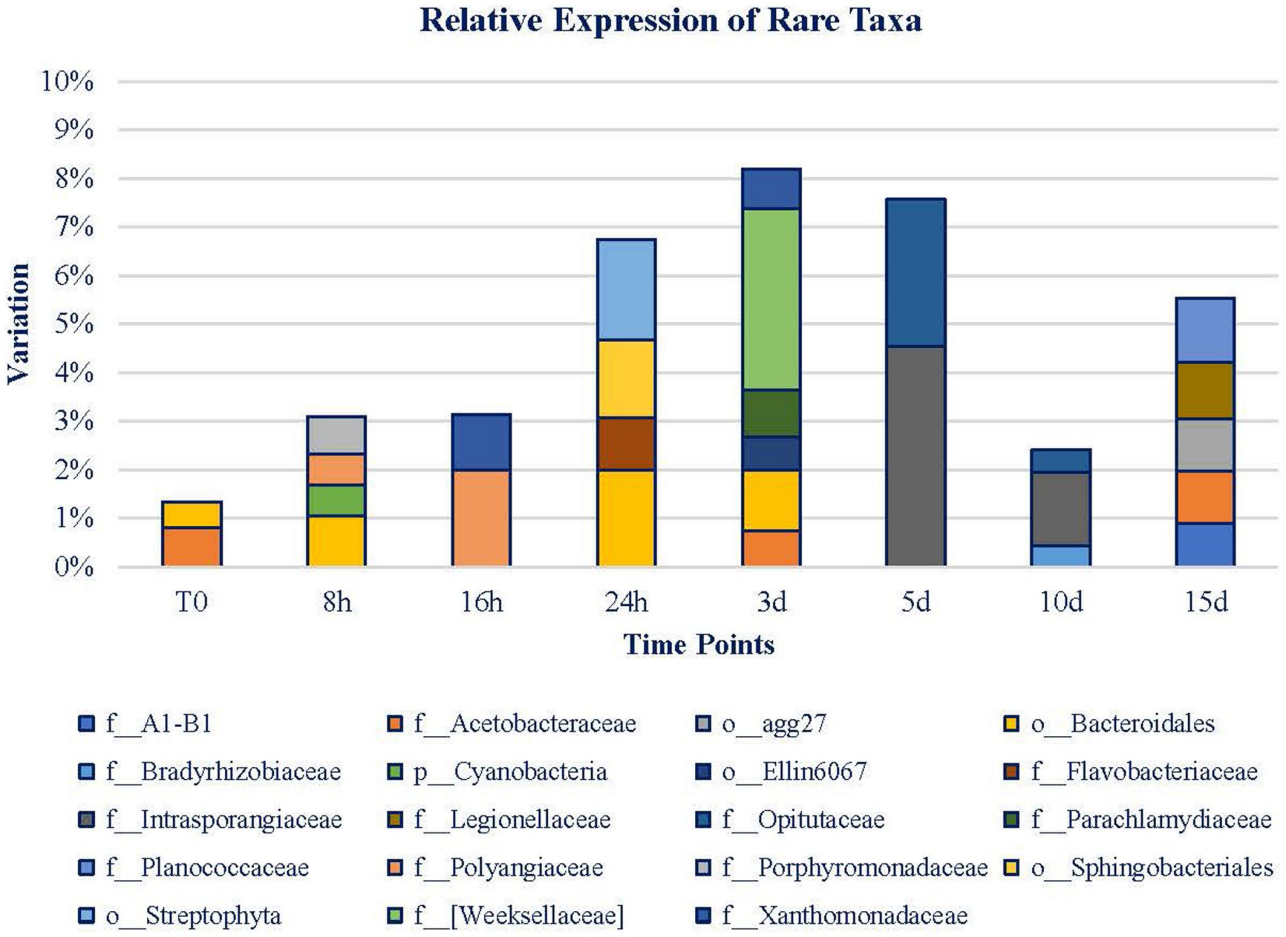
Rare taxa (<1% relative expression). Legend is ordered from left to right and up to down in alphabetical order.

Rare taxa (expression < 1%; Figure 8) only represented on average 1.34% of the diversity in the expression of the original mat. The relative expression of rare taxa increased until day 3 when they reached 8.1%, from that point on, the relative expression declined until day 10. On day 15, rare taxa recovered again. Noticeably, throughout T0 to 3 d, the microbial mat is more diverse, but throughout 5 to 15 d, expressing taxa are more conserved, and 41.66% of these taxa’s expression belongs to *Bacteroidetes* Phylum. The rest of them belong to *Actinobacteria*, *Chlamydiae*, *Cyanobacteria*, *Firmicutes*, *Gemmatimonadetes*, *Planctomycetes*, *Proteobacteria,* and *Verrucomicrobia* Phyla. *Bacteroidetes* Phylum is only noticed during the first 3 d of exposure, as well as the taxa associated with *Cyanobacteria* and *Chlamydiae* Phyla. From 5 d on, *Actinobacteria*, *Firmicutes*, *Gemmatimonadetes*, *Planctomycetes,* and *Verrucomicrobia* Phyla are detected. *Proteobacteria* is the only phylum whose expression is maintained during the whole experiment.

On a short-term basis, the *Porphyromonadaceae* and *Polyangiaceae* families and *Bacteroidales* Order are the ones that draw attention. At 16 h, *Polyangiaceae* was maintained, and *Xanthomonadaceae* gained relevance. At 24 h, the total abundance had almost reached 7%. In this case, *Flavobacteriaceae*, *Sphingobacteriales,* and *Bacteroidales* are of relevance. Although *Bacteroidales* Order is the most relevant taxa (among the rare ones) at this point, from day 3, it is no longer significant. *Weeksellaceae*, *Parachlamydiaceae*, *Acetobacteraceae,* and *Xanthomonadaceae* families are the remaining taxa representing 10%. Henceforth, the variety of taxa decreased, and the rare fraction of the mat became more stable. More than 37% of these taxa belong to the *Proteobacteria* Phylum. Meanwhile, *Bacteroidetes* Phylum expression is no longer detected. At 5 d, the level of rare taxa is slightly lower than on day 3, and it is represented by the *Opitutaceae* and *Intrasporangiaceae* families. These families decline for 5 more days while *Bradyrhizobiaceae* gains relevance and *Opitutaceae* maintains its level of expression. Henceforth, rare taxa increase their presence again, and new identities, *Legionellaceae*, *Planococcaceae,* and *Acetobacteraceae,* are highlighted at 15 d and occupy almost 6% of the mat. Among all of the detected taxa, *Acetobacteraceae* is the only one noticeable during the whole experiment, in the original mat, in the 3- and 15-d samples. As it can be noticed from Figure 8, the first range of time is characterized by the presence of 12 taxa whose abundance is low. However, the second range of time is marked by the presence of fewer taxa (8) but of higher abundance than in the first period.

## 4 Discussion

Although Antarctica is considered one of the best analogs of what planet Mars has on Earth, the conditions the cyanobacterial mat had to deal with in this experiment are still different from its conventional Antarctic environment. The cyanobacterial mat came from an environment poor in CO_2_ and rich in N_2_ (78%) and O_2_ (21%) and was introduced in an atmosphere with only the oxygen produced by the oxygenic primary producers, which in fact could be similar to the environment in Antarctic ponds under the ice (Jungblut et al., 2016). The community also had to face the low pressure in the chamber, 95–103 kPa in Byers vs. 0.1–2 kPa in MARTE. Low pressures such as the ones existing on Mars could have severe consequences on microbial communities. For example, low pressures can increase membrane fluidification, and it could result in the disorder of translocation complexes, ion transporters, or H+ pumps, resulting in metabolic impairments (Schwendner and Schuerger, 2020). The temperature in Byers Peninsula is similar to the values of Mars during the summer season, so this would not be a problem for the microbial community. However, humidity and radiation could also be a challenge for the microorganisms. Although during summer, mats are usually submerged (100% humidity), after this period, they can face dewatering phases in which humidity is around 90%, reaching in some cases extreme dehydration (20%). In the case of Mars, humidity is around 35%, which is at its maximum. The radiation case is similar; while Byers receives around 1,360 W/m^2^ of annual radiation, Mars receives around 550 W/m^2^ annually. The UV radiation would be possibly the main reason for the damage that occurred to this phylum because it can cause severe harm to the photosynthetic machinery (Rastogi et al., 2014), but also to DNA/RNA biomolecules and both protein and lipid structures, alterations in membrane permeability, N_2_ fixation, and CO_2_ uptake that affect the rest of the community as well. Thus, aerobic organisms could be at risk if cyanobacteria and other minoritarian oxygenic photosynthesizers are not able to produce enough oxygen due to the effects of UVR. However, cyanobacteria present some strategies that can be useful to bear UV radiation. At the cellular level, these mechanisms include the enforcement of antioxidant systems, the synthesis of extracellular polysaccharides, the synthesis of UV-absorbing/screening compounds, the expression of heat shock proteins (Hsps), the repair and resynthesis of damaged DNA and proteins, or *de novo* protein synthesis (Rastogi et al., 2014). At the community level, the avoidance of UV-induced damage can occur by migration or mat formation (Quesada and Vincent, 1997).

The results obtained from the sequencing analysis demonstrate that conditions in the MARTE chamber exert a marked influence on the community’s expression of the Antarctic cyanobacterial mat. The active community structure of the mat changed with the exposure to Martian conditions, but apparently after a period of exposure, the community expression returned back to some similar expression diversity than that was observed at the beginning of the experiment. Ecological analyses revealed that the biological activity of the mat is dominated mainly by six taxa, assigned to the *Pseudanabaenaceae*, *Comamonadaceae*, *Clostridiaceae*, *Frankiaceae*, *Nannocystaceae,* and *Chamaesiphonaceae* families. The relative dominance of the expression shows what looks like a shift from six very dominant taxa to a more balanced situation, involving a larger diversity in terms of relative expression.

According to Figures 2–4, Martian conditions may cause significant changes on the mat on a short-term scale. The community structure of the mat is progressively altered during the first 5 d of exposure. However, between 5 and 15 d, the mat seemed to return to a similar state of expression than before the exposure to MARTE conditions.

Changes in both ASV composition and diversity through time (Figures 2, 3) may indicate differential effects on the elements of the mat. Both the ASV richness and diversity index tend to increase during the first 16 h of exposure. At 24 h and 3 d, both variables showed a decrease in values compared to the initial ones. From day 5 of exposure, there is an apparent continuous change tending to stabilize at the end of the exposure period (15 d) to a status similar to the initial.

The variation in the quantity of active taxa during the exposure period might be the result of an adaptation process of the components of the community to the Martian conditions. While in the first increasing period, the community is still bearing the effects of Martian conditions, in the second one, it changes the structure to a more stable one (more time of exposure is necessary to induce changes in the community).

The 11 dominant taxa (relative expression >1%) belonged to four different phyla. Some families of both *Actinobacteria* and *Proteobacteria* experience negative global effects, and some others benefit from the exposure to the Martian conditions. On the other hand, the genera from *Cyanobacteria* suffered generalized negative global effects, although the opposite effect could be assumed for *Firmicutes* Phylum. Microorganisms belonging to this group are mainly Gram-negative, usually aerobic or facultative aerobes. *Clostridiaceae* and *Intrasporangiaceae* families are the only Gram-positive organisms, with *Clostridiaceae* being the only obligate anaerobe taxon. Some cases, such as the *Sphingomonadaceae* family and *Phycisphaerales* order, are facultative anaerobes. In microbial mats, strong oxyclines typically appear (Jungblut et al., 2016) with strong anaerobic conditions at the bottom and aerobic conditions on top during the daylight due to photosynthetic activity, or even supersaturated oxygen concentrations at the midlayers.

In addition, these 11 dominant taxa are usually motile (Supplementary Table 1). This ability gives them the possibility to find the most optimal layer within the microbial mat. Of 11, 4 taxa use light as an energy source (3 cyanobacteria and 1 proteobacteria): *Pseudanabaenaceae*, *Chamaesiphonaceae*, *Phormidiaceae,* and some species of *Comamonadaceae*, but other metabolisms are also found in these taxa.

The taxa that benefitted from Martian conditions were *Comamonadaceae*, *Clostridiaceae*, *Phormidiaceae,* and *Sphingomonadaceae* families. Disadvantaged taxa were *Pseudanabaenaceae* family, *Myxococcales* order, *Chamaesiphonaceae*, *Pseudomonadaceae* families, and *Phycisphaerales* order. *Frankiaceae* and *Nannocystaceae* families show no defined behavior toward Martian conditions.

Both *Comamonadaceae* and *Clostridiaceae* families seem to benefit under Martian conditions. *Comamonadaceae* increases its presence to almost 15% on day 15. Their initial decrease may be due to the decline of the oxygen levels due to the detriment of *Cyanobacteria* taxa. However, its recovery from 10 d onward may be due to other reasons, such as the wide variety of metabolism of the identified genus of *Comamonadaceae* family, such as *Rhodoferax* and *Leptothrix*. *Rhodoferax* is involved in carbon fixation while *Cyanobacteria* taxa are declining, as it appears precisely at 16 h and 3 d. In the case of *Leptothrix*, it has a chemoorganotrophic metabolism that can exploit the organic matter delivered from the death of not-so-well-adapted organisms. However, its most important characteristic is the filamentous structure, which can serve as the structure of the mat itself in addition to the one designed by filamentous cyanobacteria (Imhoff, 2006; Hiraishi and Imhoff, 2015; Willems and Gillis, 2015). In the case of *Clostridiaceae,* its wide variety of metabolisms and its movement capacity, together with the possibility of endospore formation, could be the reason behind the maintenance of this family through time. Indeed, organisms belonging to *Firmicutes* Phylum count on several peptidoglycan layers, which would be involved in resistance to desiccation (Nóbrega et al., 2021).

In contrast to the previous taxa, *Pseudanabaenaceae* family has not benefited from the Martian conditions, but it can survive through the whole experiment. *Leptolyngbya* genus would be able to bear Martian conditions such as desiccation but would perish facing UV radiation, presumably due to its inability to move (Castenholz et al., 2015a,b), although other avoidance strategies have been described in microbial mats (Quesada and Vincent, 1997). Unlike *Leptolyngbya*, *Pseudanabaena* can move by gliding, so this could be helpful to avoid UV radiation and survive exposure to Martian conditions. The resistance of the *Leptolyngbya* genus could be related to other kinds of mechanisms, such as antioxidant mechanisms to endure oxidative stress (Rastogi et al., 2014). Thus, the compendium of all, or part, of these mechanisms, together with the synthesis of extracellular polysaccharides by cyanobacteria, would allow the entire community to survive.

*Myxococcales* order does not greatly vary by its activity level but varies for its increase on day 5. They have in principle the ability to develop fructiferous bodies and to move by gliding so that they could find a place in the mat with the most favorable conditions. In addition, they may feed from insoluble organic substances (Döring and Myxococcales, 2022), occupying a unique ecological niche that would sustain them.

In total, the 19 rare taxa (relative expression is <1%) are associated with 9 different phyla whose expression is detected in remarkable points of the experiment. The initial rare taxa *Acetobacteraceae* and *Bacteriodales* are complemented by *Porphyromonadaceae* and *Polyangiaceae* in the first hour of exposure, and the four of them being Gram-negative organisms and both are aerobes and anaerobes, with saccharolytic-fermentative and proteolytic-bacteriolytic metabolisms, respectively. These organisms may obtain benefits from the organisms that were damaged by the Martian conditions. Then, other rare taxa become more prominent, likely making use of the new conditions after the community changes and intervening in their metabolic capabilities and their motility abilities.

After 3 d of exposure, the rare taxa reach approximately 8% of expression in the mat, and there are more diversities among them in terms of family than at the beginning. Some of them, such as *Weeksellaceae*, are saprophytic organisms (Bernardet, 2015). *Acetobacteraceae* was also detected indicating the potential production of acidic compounds in the aftermath of the decomposition of the organic matter (Sievers and Swings, 2015). Only 2 d later, on day 5, a change in the succession of rare taxa was found. Families not noticed before were active, and these exhibited higher expressions than the ones from the previous time samples. Among them, Gram-negative anaerobes such as *Opitutaceae* and Gram-positive organisms such as *Intrasporangiaceae* increased their proportion. Toward the end of the experiment, at day 10, although both families were still noticed, the frequency of rare taxa dropped to the initial frequencies, with an ulterior increase.

The results of this study reveal what would be the succession occurring in an Antarctic cyanobacterial mat if it had been placed in a spot similar to Mars’s surface. The expression of thinner filamentous cyanobacteria (such as *Pseudanabaenaceae*) is affected by the Martian conditions. Therefore, as they have been proposed as the architects (de los Ríos et al., 2004) of microbial mats due to their filamentous structure, if their cell walls disappear, then the physical structure of the microbial mat might be compromised. However, this could be a transitory state, since after 5 d of exposure, the expression of this structural group recovers partially (as in the case of *Pseudanabaenaceae*) or totally (in the case of *Chamaesiphonaceae* and *Phormidiaceae*). The evolution of the mat shows that taxa motility would be very relevant in a Mars environment since the organisms can find the most favorable locations. This would be the case of cyanobacteria escaping from the UV radiation. It is remarkable how Gram-positive organisms, *Clostridiaceae* among the dominant taxa and *Intrasporangiaceae* among the rare taxa, increase their presence during the experiment. The diversity of taxa and associated metabolisms, as well as the redundancy of some key metabolisms, might be necessary to keep the functioning of the microbial mat even under the most stringent conditions as the Martian environmental conditions. Considering these results, if the mat had been exposed for longer periods of time, we speculate that the community succession is dependent on the complete recovery of cyanobacterial taxa, which in fact would rely on the resilience against the UVR impact; however, this hypothesis requires further research.

At the beginning of this study, we assumed that the Martian conditions should not have an effect on the structure of the community. This way, the mat, as a community, should be capable of surviving the situation. Our results showed that, while some taxa have been especially affected by the exposure to Martian conditions, others may have even increased their biological activity.

We speculate that it is the physical structure of the microbial mat itself and not only the microbial community structure that makes this microecosystem resilient to the extremely harsh conditions of this experiment. It is perhaps the diverse metabolic features of the organisms conforming to this ecosystem that makes it a potential candidate as a *Martian terraformis* element, as it was suggested by Friedmann (Fogg and Hiscox, 2001).

## 5 Conclusion

Although most taxa experienced a decline and recovered in their activity levels during the exposure to Martian conditions, the changes in the mat’s active community structure enabled the apparent increase in the frequency of active taxa that were previously minority. This phenomenon would be due to the impact of Martian conditions that have upon the entire community but to the ability of cyanobacterial taxa to endure these extremely harsh conditions. Perhaps, oxic/anoxic conditions affect, in general terms, the expression of the community. During the first stages of the exposition, it looks like there is a trend to a reduction of the activity in aerobic taxa, and this may exert an influence on the environmental conditions from oxic to anoxic, perhaps caused by UVR effects upon photosynthetic machinery. Then, it seems that cyanobacterial taxa could acclimatize to Martian conditions, perhaps returning to an oxic environment. However, this hypothesis requires further research and experimental data acquisition.

Apparently, the Antarctic cyanobacterial mat from Byers Peninsula would be able to survive and perhaps prosper in the Martian environment, due to its architecture but also to the complex and diverse community that forms the consortium.

## Data availability statement

The datasets presented in this study can be found in online repositories. The names of the repository/repositories and accession number(s) can be found in the article/Supplementary material.

## Author contributions

IM-A: Methodology, Writing – original draft. JS: Conceptualization, Funding acquisition, Methodology, Writing – review & editing. EC: Conceptualization, Methodology, Writing – review & editing. AQ: Conceptualization, Funding acquisition, Methodology, Supervision, Validation, Writing – review & editing.
