# Supplementary figures and images for: Survival of an Antarctic cyanobacterial mat under Martian conditions
Source: Front Microbiol. 2024 Apr 5;15:1350457. doi: 10.3389/fmicb.2024.1350457 (PMC11027934; doi:10.3389/fmicb.2024.1350457)

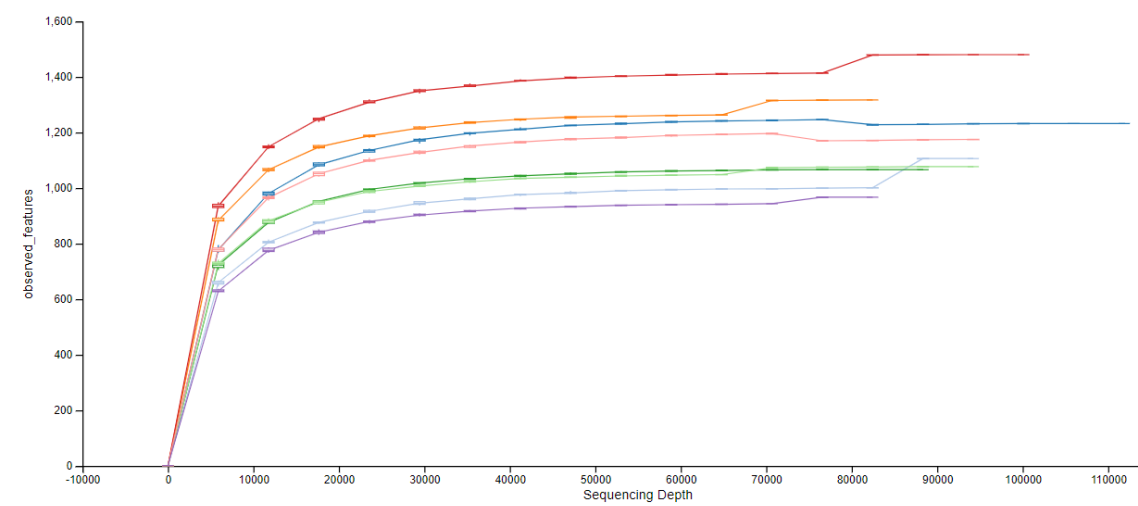

| Sample | T0    |       | 8h     |        | 16h     |         | 24h     |         | 3d     |        | 5d     |        | 10d     |         |
|--------|-------|-------|--------|--------|---------|---------|---------|---------|--------|--------|--------|--------|---------|---------|
|        | C_S22 | C_S30 | 8h_S23 | 8h_S31 | 16h_S24 | 16h_S32 | 24h_S25 | 24h_S33 | 3d_S26 | 3d_S34 | 5d_S27 | 5d_S35 | 10d_S28 | 10d_S36 |
| Colour |       |       |        |        |         |         |         |         |        |        |        |        |         |         |

**Figure S1.** *Rarefaction curve*

Supplement: Supplementary file 1 [file Data_Sheet_1.PDF]
