# Supplementary material for: Survival of an Antarctic cyanobacterial mat under Martian conditions
Source: Front Microbiol. 2024 Apr 5;15:1350457. doi: 10.3389/fmicb.2024.1350457 (PMC11027934; doi:10.3389/fmicb.2024.1350457)
Supplement: Supplementary file 2 [file Table_1.pdf]

Suplementary Material Table 1. Relevant information of dominant taxa - Families and Order*. If genus level has been identified, it is stated. Gram classification is stated either positive (+), or negative (-).

Dominant taxa		***Gram***	***Respiratory***	***Main metabolism***	***Motility***	***Other characteristics***	***References***
Family	***Genus***						
Pseudanabaenaceae	*Leptolyngbya*	-	Aerobic	Photoautotrophic	Slow gliding/non-motility	Filamentous	*Castenholz et al., (2015a)*
	Pseudanabaena	-	Aerobic	Photoautotrophic	Gliding	Filamentous	*Castenholz et al., (2015b)*
Comamonadaceae	*Leptothrix*	-	Strictly aerobes	Chemoorganotrophic	Single polar flagellum/bipolar tufts (1–5 flagella)	Filamentous structure	*Willems & Gillis, (2015).*<br><br>*Leptothrix | SpringerLink*
	Polaromonas	-	Strictly aerobes	Chemoorganotrophic /facultatively chemolithotrophic	Single polar flagellum	Gas vacuoles	*Willems & Gillis, (2015).*
	Rhodoferax	-	Strictly aerobes<br><br>Some of them can breathe nitrates	Photoheterotrophic/Anoxygenic photosynthesis/Fermentation	Polar tufts	No endospores<br><br>Anaerobically growth in light and aerobically growth in (in full oxygen tension environment)	*Willems & Gillis, (2015).*<br><br>*Hiraishi & Imhoff, (2015)*<br><br>*Imhoff (2006).*
	Methylibium	-	Strictly aerobes	Chemoorganotrophic/ Facultatively chemolithotrophic	Single polar flagellum/bipolar tufts (1–5 flagella)	-	*Willems & Gillis, (2015).*

	Paucibacter	-	Strictly aerobes	Chemoorganotrophic/ Facultatively chemolithotrophic	Single polar flagellum/bipolar tufts (1–5 flagella)	-	*Willems & Gillis, (2015).*
Clostridiaceae		+	Strictly anaerobes (usually)	Heterotrophs/glycolytic/ saccharolytic /peptolytic/ chemolithoautotrophic/ fermentation (organic acids and alcohols products)	Peritrichous flagellation	Endospore formation	*Wiegel (2015).* *Spring et al., (2003).*
Myxococcales		-	-	Insoluble-organic substances	Gliding	Fructiferous bodies	*Döring, (2022)*
Frankiaceae		+	Aerobic/microaerophilic	Chemoorganotrophic	No motility	N_2 fixation	*Normand, (2014)*
Chamaesiphonaceae		-	Aerobic	Photoautotrophs	-	Oxygen producer	*Encyclopedia of Life. Chamaesiphonaceae [en línea] Disponible en: https://eol.org/pages/3230*
Phormidiaceae	*Phormidium*	-	Aerobic	Photoautotrophs	Moving capacity	Filamentous structure Vertical migration against UV light	*Bellinger & Sigee, (2015).* *Pattanaik et al., (2007)*
Nannocystaceae		-	Aerobic	Proteolytic–bacteriolytic	Swarming/Gliding	Mesophilic	*Reichenbach (2015).*

						Estuarine and marine environments	*Garcia & Müller (2014)*
Pseudomonadaceae		-	Aerobic/Anaerobic	Chemoorganotrophic	Polar flagella	Different environments	*Palleroni, (1981).*
Sphingomonadaceae	*Zymomonas*	-	Facultatively anaerobic/ strictly anaerobic (some strains)	Chemoorganotrophic<br><br>Glucose and fructose fermenters	Non-motility/1-4 lophotrichous flagella	No spore formation<br><br>Optimum growth at 25-30 ºC, slow at 15 ºC, no growth at 4 ºC	*Yabuuchi & Kosako (2015).*<br><br>*Sahm (2006)*<br><br>*Sprenger & Swings (2014)*

References List

Bellinger EG, Sigee DC, eds. Freshwater Algae: identification, enumeration and use as bioindicators. 2nd Edition. Chichester: Wiley Blackwell, 2015

Castenholz, R. W., Rippka, R., Herdman, M., and Wilmotte, A. (2015a). “Form-Leptolyngbya” in *Bergey's manual of systematics of Archaea and Bacteria* (Chichester, UK: John Wiley & Sons, Ltd) In: (eds) M.E. Trujillo, S. Dedysh, P. DeVos, B. Hedlund, P. Kämpfer, F.A. Rainey and W.B. Whitman)

Castenholz RW, Rippka R, Herdman M, et al, Form- Pseudanabaena.. In Bergey's Manual of Systematics of Archaea and Bacteria, edited by John Wiley & Sons, Ltd, Chichester, UK, 2015b. doi: 10.1002/9781118960608.gbm00444. Available in: <https://onlinelibrary.wiley.com/doi/abs/10.1002/9781118960608.gbm00444>. In: (eds) M.E. Trujillo, S. Dedysh, P. DeVos, B. Hedlund, P. Kämpfer, F.A. Rainey and W.B. Whitman

Döring M. Myxococcales Tchan et al., 2022. Available online at https://www.gbif.org/ru/species/144093638.

Encyclopedia of Life. Chamaesiphonaceae [online] Available at: https://eol.org/pages/3230 → Encyclopaedia of Life. Chamaesiphonaceae. No year. Available online at https://eol.org/pages/3230.

Garcia, R. & Müller R. The Family Nannocystaceae. In The Prokaryotes – Deltaproteobacteria and Epsilonproteobacteria, edited by Springer-Verlag Berlin Heidelberg. 2014 doi: 10.1007/978-3-642-39044-9_305.

Hiraishi A, Imhoff JF. Rhodoferax. In Bergey's Manual of Systematics of Archaea and Bacteria, edited by John Wiley & Sons, Ltd, Chichester, UK, 2015. doi: 10.1002/9781118960608.gbm00951. In: (eds) M.E. Trujillo, S. Dedysh, P. DeVos, B. Hedlund, P. Kämpfer, F.A. Rainey and W.B. Whitman)

Imhoff JF. The Phototrophic Beta-Proteobacteria. In The Prokaryotes, edited by Springer New York, New York, NY, 2006;pp. 593-601 doi:10.1007/0-387-30745-1_25.

Normand P. The Family Frankiaceae. In The Prokaryotes edited by Springer Berlin Heidelberg, Berlin, Heidelberg, Nov 01, 2014;pp. 339-356. doi:10.1007/978-3-642-30138-4_183.

Palleroni NJ. Introduction to the Family Pseudomonadaceae. In: Starr, M.P., Stolp, H., Trüper, H.G., Balows, A., Schlegel, H.G. Ed: The Prokaryotes. Springer, Berlin, Heidelberg. 1981 https://doi.org/10.1007/978-3-662-13187-9_58

Pattanaik B., Schumann, R., Karsten, U. Effects of ultraviolet radiation on cyanobacteria and their protective mechanisms. In: Cellular Origin, Life in Extreme Habitats and Astrobiology: Algae and Cyanobacteria in Extreme Environments. Ed: Seckbach, J. 2007: Springer, 29-45

Reichenbach H. Nannocystaceae fam. nov. In Bergey's Manual of Systematics of Archaea and Bacteria, edited by John Wiley & Sons, Ltd, Chichester, UK, 2015 doi: 10.1002/9781118960608.fbm00206. In: (eds) M.E. Trujillo, S. Dedysh, P. DeVos, B. Hedlund, P. Kämpfer, F.A. Rainey and W.B. Whitman)

Sahm H. The Genus Zymomonas. In The Prokaryotes, edited by Springer New York, New York, NY, 2006;pp. 201-221. doi:10.1007/0-387-30745-1_10.

Sprenger GA, Swings J. Zymomonas. In Bergey's Manual of Systematics of Archaea and Bacteria, edited by John Wiley & Sons, Ltd, Chichester, UK, 2014. doi: 10.1002/9781118960608.gbm00925. In: (eds) M.E. Trujillo, S. Dedysh, P. DeVos, B. Hedlund, P. Kämpfer, F.A. Rainey and W.B. Whitman)

Spring S, Merkhoffer B, Weiss N, et al. Characterization of novel psychrophilic clostridia from an Antarctic microbial mat: description of Clostridium frigoris sp. nov., Clostridium lacusfryxellense sp. nov., Clostridium bowmanii sp. nov. and Clostridium psychrophilum sp. nov. and reclassification of Clostridium laramiense as Clostridium estertheticum subsp. laramiense subsp. nov. Internat*ional* Journal of Systematic and Evolutionary Microbiology 2003;53(4): 1019-1029. doi: 10.1099/ijs.0.02554-0.

Wiegel J. Clostridiaceae. In Bergey's Manual of Systematics of Archaea and Bacteria, edited by John Wiley & Sons, Ltd, Chichester, UK, 2015. doi: 10.1002/9781118960608.fbm00129. In: (eds) M.E. Trujillo, S. Dedysh, P. DeVos, B. Hedlund, P. Kämpfer, F.A. Rainey and W.B. Whitman)

Willems A, Gillis M. Comamonadaceae. 2015 In Bergey's Manual of Systematics of Archaea and Bacteria, edited by John Wiley & Sons, Ltd, Chichester, UK, 2015. doi: 10.1002/9781118960608.fbm00182. In: (eds) M.E. Trujillo, S. Dedysh, P. DeVos, B. Hedlund, P. Kämpfer, F.A. Rainey and W.B. Whitman)

Yabuuchi & Kosako (2015a). → Yabuuchi E, Kosako Y. Sphingomonadales ord. nov. In Bergey's Manual of Systematics of Archaea and Bacteria, edited by John Wiley & Sons, Ltd, Chichester, UK, 2015. doi: 10.1002/9781118960608.obm00075. In: (eds) M.E. Trujillo, S. Dedysh, P. DeVos, B. Hedlund, P. Kämpfer, F.A. Rainey and W.B. Whitman)
